# A vision for inclusion

**Published:** 2013

**Authors:** Victor John Cordeiro

**Affiliations:** Advocacy Coordinator: World Blind Union, Bangalore, India.

**Figure:**
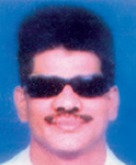
Victor John Cordeiro

Nandini comes from a remote village in Karnataka, in the south of India, and was blind at birth as a result of retinitis pigmentosa. She has faced many challenges – including attempts to exclude her from school – because of her visual disability. In high school, Nandini made contact with the National Association for the Blind (NAB India) and local self-advocacy groups of people who are blind or visually impaired.

Once she left school, Nandini was asked to become regional coordinator for the All India Confederation of the Blind (AICB). AICB is a coordinating body made up of 22 grass-roots and state-level associations or organisations working with people who are blind, and is also a member of the World Blind Union. Nandini's remit was to promote the empowerment of women and girls who are blind or have impaired vision who, like her, faced discrimination, injustice, abuse, and violence.

**‘Nandini faced many challenges, including attempts to exclude her from her school because of her visual disability’**

Nandini was later promoted to national coordinator of AICB, and her efforts resulted in policy changes to make voting in elections accessible for people with impaired vision, as well as improvements in their ability to access education and earn a living. She presented papers on related issues to international forums organised by the World Blind Union.

Nandini now works with ActionAid, where she is in charge of promoting the rights of women, including those with disabilities. She writes, composes, and sings revolutionary songs to encourage women in their struggle for equity and justice.

Nandini's contact with local self-advocacy groups, NAB India, AICB and other such disabled people's organisations, have significantly contributed in shaping her attitudes, perspectives and building capacity to deal with the challenges which blind or partially sighted women and girls face in day-to-day life.

**Figure:**
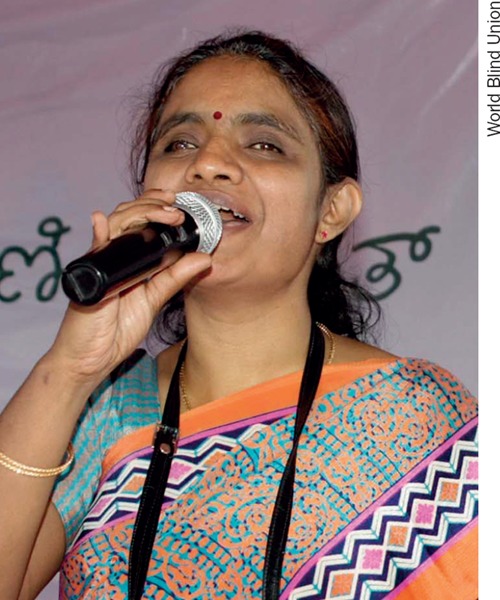
Nandini uses music to deliver her message of empowerment. INDIA

Nandini says her association with these organisations has boosted her morale, provided her with exposure to a wide range of issues, and prepared her as an activist in order to initiate systematic and organised struggle. This has enabled her to address injustice and exclusion in an organised and systematic manner.

**Disabled people's organisations: a valuable link for eye care workers**

Most countries have disabled people's organisations (DPOs). These organisations are founded and run by people with disabilities, for people with disabilities.

Local DPOs tend to be linked to organisations at provincial/district level, which are in turn linked to DPOs at national and eventually international level. These organisations exist for disabilities in general or for specific impairments, such as visual impairment and blindness. The World Blind Union is one example of an international DPO for people with vision impairment.

DPOs can act as a valuable link for eye care workers. A DPO can connect people with vision loss to other DPO members, as well as to community organisations that support people with disabilities. It is therefore good practice for eye care workers to make contact with local DPOs.

There are several ways in which DPOs can help, e.g. raising awareness, advocacy, disability management and improving disability inclusion within organisations.

DPOs can help the eye clinic by:

- Giving practical advice on how to make a clinic more accessible and user-friendly, whether for people with visual, hearing, intellectual or mobility impairments
- Providing training on disability awareness to encourage positive perceptions and dispel myths about disability among clinic staff
- Encouraging referral of people with impaired vision for ongoing eye screening, and of people with other impairments for regular checkups
- Encouraging referral of people with impaired vision to the DPO, and for other rehabilitation or education opportunities
- Actively seeking people with disabilities to attend screening
- Disseminating promotional messages about eye health to ensure that all DPO members have access and can further promote this information to other community members with disabilities.

DPOs can help patients by:

- Advocating for them, and training them in self-advocacy skills
- Mentoring people with a permanent impairment in the development of independent living skills
- Creating connections to community-based disability and other services
- Creating connections to other people with disabilities.

**Guidelines for referral**

- Develop clear two-way referral pathways and effective referral systems, including the appointment of a key staff member to oversee referrals and to maintain an up-to-date list of disability services and DPOs.
- Improve awareness of the presence and roles of disability services and DPOs among all staff.
- Connect with local CBR services, other disability services, inclusive education options and DPOs to improve knowledge of their role and formalise referral processes.
- For women with disabilities, determine whether there is a preference to meet with a female disability service or DPO member, and ensure this is clearly indicated on the referral.

Compiled by David Lewis and Joanne Webber

